# Successful management of trachea stenosis with massive substernal goiter via thacheobronchial stent

**DOI:** 10.1186/1749-8090-8-212

**Published:** 2013-11-15

**Authors:** Meihua Piao, Ye Yuan, Yanshu Wang, Chunsheng Feng

**Affiliations:** 1 Department of Anesthesiology, the First Hospital of Jilin University, Changchun 130021, Jilin, PR China

**Keywords:** Tracheal stenosis, Tracheobronchial stent, Airway management, Substernal goiter

## Abstract

A case of 65 year-old Chinese male patient with severe tracheal stenosis due to a massive substernal goiter, is presented. MRI and CT scan revealed that the massive substernal goiter was 9.3 × 6.1 × 4.7 cm in size, displacing the trachea and adjacent large vessels to the patient’s right contributing to severe intrathoracic trachea compression up to 6 cm in length and the narrowest caliber of the trachea only 3.0 mm in diameter. To the best of our knowledge, optimal airway management for the massive substernal goiter resection was considered to be temporary tracheobronchial stent placement pre-operation.

## Background

Total obstruction of the airway is a life-threatening complication in patients with severe tracheal stenosis caused by substernal goiter, which is generally defined as a thyroid mass that has 50% or more of its volume located below the thoracic inlet [1], although retrosternal is probably a more precise term. Substernal goiter may be detected incidentally on chest x-ray or CT scan or found because of obstructive symptoms such as dyspnea, wheezing, or cough. The most common obstructive symptom is exertional dyspnea, which is present in 30 to 60 percent of cases, and usually occurs when the tracheal diameter is less than 8 mm. The airway obstruction can occur during induction of anesthesia, extubation, surgical resection, or simply by a change of posture. How to establish a patent airway peri-operation is of crucial importance and great challenge to the anesthesiologist. Herein, a case of severe tracheal stenosis due to external compression by a massive substernal goiter, is presented. A temporary endoluminal self-expandable metallic stent (SEMS) was acquired successfully pre-operation which was considered the optimal management to improve the airway obstruction.

## Case presentation

### Case report

A 65-year old, 57 kg weighed Chinese male patient came to the outpatient clinic complained of anterior neck swelling and dyspnea. He started to notice his anterior neck swelling five years ago and it was progressively increasing in size. During the last four months he developed aggressive dyspnea, and in the recent two weeks he presented blue lips (cyanosis) and obvious obstructive symptoms including shortness of breath, inspiratory and expiratory stridor in awake, dyspnea and could not lie in supine completely at all. He was forced orthopnea at daytime and hardly could he fall asleep at night except for turning in left lateral decubitus for a while which he felt better for breathing, but often awaked due to dyspnea as he fell asleep. He denied of any underlying medical illness.

### Examination

**Figure 1:**
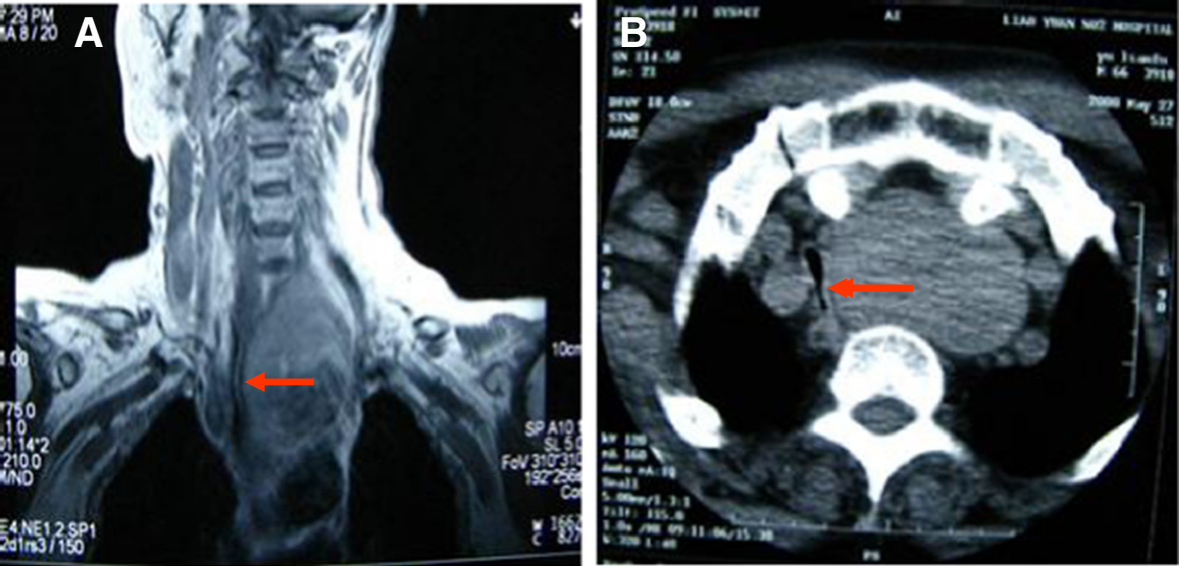
Magnetic resonance imaging (MRI) (A) and computed tomography (CT) (B) demonstrated a massive elliptical substernal goiter, displacing the trachea and adjacent large vessels to the patient’s right, contributing to severe intrathoracic tracheal compression up to 6 cm in length and the narrowest caliber of the trachea (arrows in light red) was about 3.0 mm in diameter.

**Figure 2:**
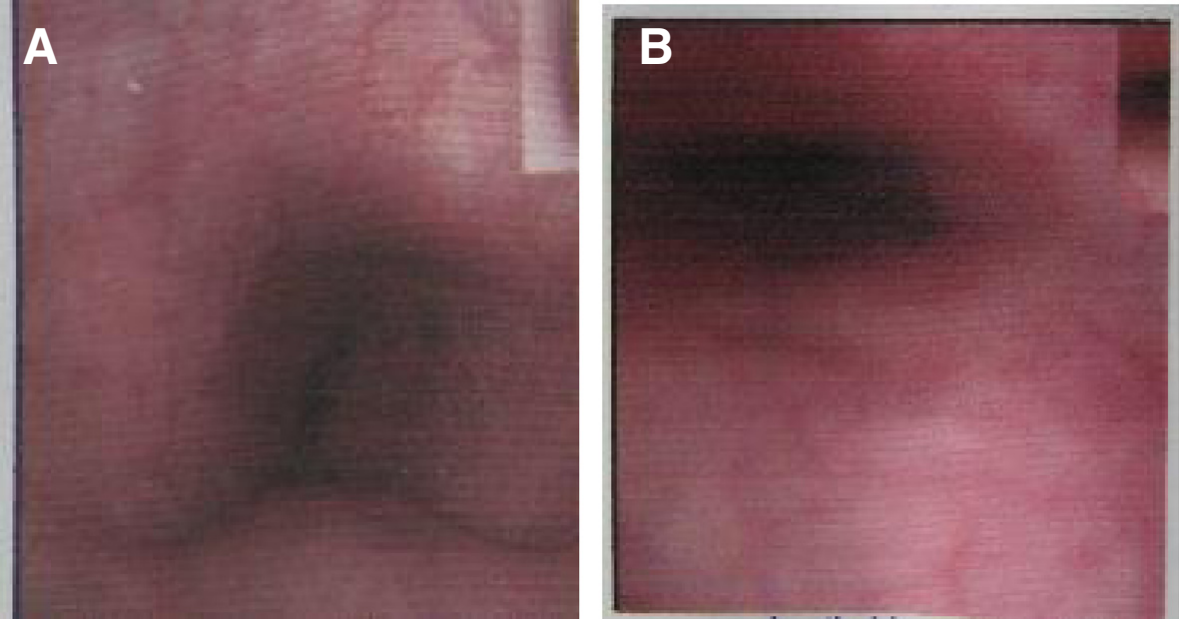
**The bronchoscopic examination through nose showed that the trachea 20 cm from the nostril was depressed (A), and the site 25 cm from the nostril was narrowed seriously (B) so that the bronchoscope of outer diameter (OD) 2.8 mm could barely go through with some resistance.** The tunica mucosa bronchiorum was smooth under bronchoscopy.

On examination, a tumor in the left lobe of thyroid with the size of 10 × 8 cm could be touched. It extended from below the chin level until the suprasternal notch and the inferior margin was not palpable. Magnetic resonance imaging (MRI) and computed tomography (CT) of the neck demonstrated a massive elliptical substernal goiter measuring 9.3 × 6.1 × 4.7 cm, displacing the trachea and adjacent large vessels to the patient’s right, contributing to severe intrathoracic trachea compression up to 6 cm in length and the narrowest caliber of the trachea only 3.0 mm in diameter (D). The trachea should run straight from the mouth/nose down to the lungs rather than being curved (outlined in light red) like it is in this picture (Figure 1). The bronchoscopic examination through nose showed that the trachea was depressed at 20 cm from the nostril, and at the level of 25 cm from the nostril it was narrowed even more seriously so that the bronchoscope of outer diameter (OD) 2.8 mm could barely go through with some resistance. The tunica mucosa bronchiorum was smooth under bronchoscopy. (Figure 2) This procedure was forced to an end because of the patient’s obvious apopnixis. Arterial blood gas analysis (ABGA) showed that pH 7.39, PaCO_2_ 42 mmHg, PaO_2_ 56 mmHg and SpO_2_ 85% with room air.

Pre-operative airway evaluation revealed Mallampati score class 2, normal thyromental distance and no limitation of neck or mandible movements. The emphasis of anesthesia was focused on airway management strategies for this patient, aimed at relieving airway obstruction peri-operation. Routine airway management such as laryngeal mask airway and endotracheal intubation was impossible for this patient as the narrowest part of the trachea lied beneath the sternum. Once the tracheal obstruction occurs, tracheostomy did not seem feasible. Thus, after meticulous consideration and discussion with the surgeons, we suggested placement of a tracheobronchial stent under topical anesthesia be the first choice for airway obstruction improvement.

### Tracheobronchial stent placement

**Figure 3:**
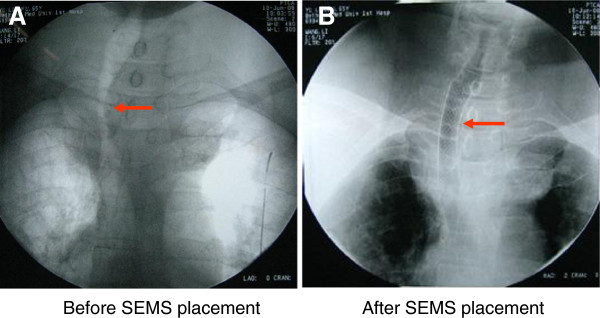
**Before placement of a self-expendable metallic stent (SEMS), the trachea was dislocated and seriously compressed by the substernal goiter (A).** But after placement of a SEMS **(B)**, the stenosis was improved significantly, the caliber of the stenotic trachea was extended to 10 mm, and the symptom of dyspnea improved immediately.

In the interventional radiology suite, topical pharyngeal anesthesia was started using a standard nebulizer filled with 3 ml of 1% tetracaine and driven by a flow of 8 L of oxygen per minute. Topical anesthesia of trachea mucosa was then performed with 3 ml of 1% tetracaine via thyrocricocentesis by coughing to confirm diffusion of anesthetic within the trachea. An endoluminal self-expandable metallic stent, 10 cm in length, is delivered over a guidewire under fluoroscopic guidance. Fluoroscopic result was immediately reviewed as soon as successful and uneventful placement of the stent, the caliber of the stenotic trachea was extended to 10 mm (Figure 3), and the obstructive symptoms improved immediately, SpO_2_ increased to 96% with room air.

The day after tracheobronchial stent placement, the patient was scheduled for thyroidectomy. On arrival at the operating room, standard monitors including noninvasive blood pressure (NIBP), electrogram (ECG), oxygen saturation (SpO_2_) and end tidal carbondioxide (ETCO_2_) were applied. Anesthesia was induced with midazolam 2 mg, fentanyl 0.3 mg, vecuronium 8 mg and propofol 120 mg, and a size of inner diameter (ID) 7.0 mm reinforced endotracheal tube (ETT) was inserted under fibroscopic guidance. Anesthesia was maintained with intravenous infusion of propofol and remifentanil. The surgical procedure was uneventful. The ventilator was weaned off 20 min after the surgery and the patient was then extubated as his consciousness recovered. The patient was observed in the post-anesthesia care unit (PACU) for one hour before he was sent to the ward. He denied of any discomfort and his SpO_2_ maintained around 95% with room air at PACU. The patient was discharged home on the seventh post-operative day and the tracheobronchial stent was removed two months later without any complication.

## Discussion

Tracheobronchial stenosis leading to flow-limiting compromise of major airway may result from a heterogeneous group of disorders including neoplasm, inflammatory disease, extrinsic compression, tracheomalacia, trauma, and strictures that can occur after intubation or surgery [2].

Narrowing of the airway lumen may produce obstructive symptoms ranging from shortness of breath to asphyxiation. Anesthetic management of a patient with a massive cervico-mediastinal goiter always gives rise to additional challenges to the anesthesiologist including difficult intubation and tracheal collapse after extubation. Depending on the severity of trachea stenosis and its location, there may be a variety of choices for peri-operative airway management such as facemask, tracheal intubation tube [3,4], laryngeal mask airway [5], tracheotomy, cardiopulmonary bypass, and extracorporeal membrane oxygenation (ECMO) [6]. But these choices seem unfit for this patient. After team consultation among thoracic, cardiovascular, thyroid surgeons and anesthesiologists, we decided to implant a temporary tracheobronchial stent with topical anesthesia under fluoroscopic guidance at first, and then perform thyroidectomy surgery with fibroscope guided reinforced ETT intubation under general anesthesia.

The anesthetic considerations for patients with a substernal goiter vary according to the individual anatomy, pathology and the proposed surgical procedure. Thus, although there are general principles of secure anesthesia for these patients, it is imperative to individualize airway management on a case-by-case basis.

Awake intubation risks endotracheal edema in this patient as repeat attempts for the ETT passing through the stenosis are inevitable somehow though. In this case, even mild edema will lead to fatal airway obstruction. We should neither choose a usual ETT (ID 7.0-8.0 mm for adult) for intubation whose narrowest caliber of the stenotic trachea was 3.0 mm in diameter nor should we choose a thinner ETT which is insufficient for ventilation.

An alternative method of airway secure is the use of emergency attempts at tracheostomy impending total airway obstruction during operation candidate or induction of anesthesia [7]. This method was ever considered in the present case. However, prophylactic tracheostomy was infeasible to relieve airway obstruction in this patient because the tracheal stenosis is far below the site of the tracheotomy orifice.

A same catastrophic situation may arise during induction of anesthesia. Relaxation of airway smooth muscle with anesthetic agents further compressed the airway, followed by inability of ventilation for this patient. What’s the worse, ETT intubation at this moment is not accessible neither, contributing to life-threatening airway obstruction.

Wang GS et al. [8] reported thyroidectomy for trachea compression under extracorporeal circulation. Since cardiopulmonary bypass and ECMO results in a systemic inflammatory response syndrome characterized by an increase in capillary permeability and edematous changes in trachea and thyroids, bleeding,acute renal dysfunction, pulmonary incompetence and higher expenses. Its adverse effects may somehow outweigh the benefits. So if it were not the optimum, it had to be the last choice.

Airway stents have been most commonly used for palliation in patients with malignant airway obstruction (MAO) such as lung and esophageal cancer, thyroid carcinoma and lymphoma that are not amenable to surgical or bronchoscopic repair [9-12]. In spite of its application and durability concerns in patients with benign disease [2,13], it has been shown increasing popularity with benign diseases such as tracheal strictures and tracheobronchomalacia due to large-sized goiter compressing over the tracheal structures for a long duration [14-16]. We reported in this case that the application of the SEMS aimed at not only improve pre-operative airway obstruction but also protect the potential airway collapse from post-operative tracheomalacia.

Placement of endoluminal stent offers a rapid and effective means of airway patency, thereby leading to dramatic improvement of symptoms and pulmonary function [17]. It has been documented that after placement of the tracheobronchial stent, dramatic improvements of life-threatening dyspnea in 70%–100% of patients with airway stenosis can be achieved immediately [18], which in turn prepare him for the surgery much better. This is also why we choose temporary SEMSs for this patient of massive thyroid goitor.

The effect of the stent in adult patients is better than in pediatric ones, because issues of trachea and pulmonary in pediatric patients grow gradually, leading to the possibility of stent removal and replacement [7]. Tracheobronchial stent is indicated in both benign and malignant airway management, but the most common indication is malignant stenosis [19-22].

It is reported that complications of airway stent occur in up to 18% of patients [23,24]. The most common and serious complications are granulation tissue formation and secretion obstruction. Other problems associated with stents include migration, malposition, infection, slippage and bleeding [25]. On the day of operation, we insisted on ETT intubation under fibroscope guidance to avoid migration or malposition of the stent. Follow-up of the patients is mandatory to prevent the formation of obstructing granulation tissue. These complications did not appear in this case possibly due to its short-term duration (only two months).

As mentioned above, tracheobronchial stent placement may be the optimal choice for the patient and protect the trachea from potential collapse due to tracheomalacia by substernal goiter’ s long-term compression after extubation. In this case, we succeeded in alleviating the tracheal obstruction via tracheobronchial stent and performed the thyroidectomy uneventfully.

## Conclusion

We herein conclude that stent placement with acceptable complications is a viable option for severe tracheal stenosis by massive substernal goiter especially for those who need surgical resection. In some patients this procedure may be the final or even optimal solution offering stable short-term or long-term airway stability.

## Consent

Written informed consent was obtained from the patient for publication of this Case report and any accompanying images. A copy of the written consent is available for review by the Editor-in-Chief of this journal.

## Abbreviations

MRI: Magnetic resonance imaging; CT: Computed tomography; SEMS: Self-expandable metallic stent; D: Diameter; OD: Outer diameter; ID: Inner diameter; ABGA: Arterial blood gas analysis; PaCO2: Arterial carbon dioxide pressure; PaO2: Arterial oxygen tension; ECG: Electrogram; PACU: Post-anesthesia care unit; NIBP: Non-invasive blood pressure; ETCO2: End tidal carbondioxide; ETT: Endotracheal tube; ECMO: Extracorporeal membrane oxygenation; MAO: Malignant airway obstruction.

## Competing interests

The authors declared that they have no competing interest.

## Authors’ contributions

MP drafted the manuscript. YY was involved in the drafting of the manuscript. YW was involved in the drafting of the manuscript. CF revised the manuscript and has given final approval of the version to be published. All authors read and approved the final manuscript.

## References

[B1] Hardy RG Bliss RD Lennard TWJ Balasubramanian SP Management of retrosternal goitres Ann R Coll Surg Engl 2009 January 91 1 8 11 19126330 10.1308/003588409X359196 PMC2752232

[B2] Raymond Thornton H Roy Gordon L Robert Kerlan K Jeanne LaBerge M Outcomes of tracheobronchial stent placement for benign disease Radiology 2006 240 1 273 282 10.1148/radiol.2401042169 16793984

[B3] Asai T Shingu K Airway management of a patient with tracheal stenosis for surgery in the prone position Can J Anaesth 2004 51 733 736 10.1007/BF03018435 15310645

[B4] Cook TM Seller C Gupta K Thornton M O’Sullivan E Non-conventional uses of the aintree intubating catheter in management of the difficult airway Anaesthesia 2007 62 169 174 10.1111/j.1365-2044.2006.04909.x 17223810

[B5] Nouraei SA Giussani DA Howard DJ Sandhu GS Physiological comparison of spontaneous and positive-pressure ventilation in laryngotracheal stenosis Br J Anaesth 2008 101 419 423 10.1093/bja/aen171 18577538

[B6] Asai T Emergency cardiopulmonary bypass in a patient with a mediastinal mass Anaesthesia 2007 62 859 860 10.1111/j.1365-2044.2007.05210.x 17635456

[B7] Ulhuda A Siddiqui KM Khan FH Emergency airway management of a patient with mediastinal mass J Pak Med Assoc 2007 57 152 154 17432024

[B8] Wang GS Lin SL Yang L Wang Z Surgical management of tracheal compression caused by mediastinal goiter: is extracorporeal circulation requisite? J Thorac Dis 2009 1 48 50 22263003 PMC3256485

[B9] Rafanan AL Mehta AC Stenting of the tracheobronchial tree Radiol Clin North Am 2000 38 2 395 408 10.1016/S0033-8389(05)70170-6 10765397

[B10] Wood DE Management of malignant tracheobronchial obstruction Surg Clin North Am 2002 82 621 642 10.1016/S0039-6109(02)00025-7 12371589

[B11] Robert MF Vito F Peter C Tracheobronchial stenting for the treatment of airway obstruction J Pediatr Surg 1998 133 304 311 10.1016/s0022-3468(98)90452-3 9498407

[B12] Rajeev P Ezzat T Slade M Sadler GP Tracheal stenting has minimal impact on survival in anaplastic thyroid carcinoma World J Surg 2013 Nov 37 11 2589 2593 10.1007/s00268-013-2173-8 23912397

[B13] Charokopos N Foroulis CN Rouska E Sileli MN The management of post-intubation tracheal stenoses with self-expandable stents: early and long-term results in 11 cases Eur J Cardiothorac Surg 2011 Oct 40 4 919 924 21316981 10.1016/j.ejcts.2010.12.042

[B14] Noppen M Stratakos G Amjadi K De Weerdt S Stenting allows weaning and extubation in ventilator or tracheostomy dependency secondary to benign airway disease Respir Med 2007 101 139 145 10.1016/j.rmed.2006.03.037 16709452

[B15] Kim WK Shin JH Kim JH Song JW Management of tracheal obstruction caused by benign or malignant thyroid disease using covered retrievable self-expandable nitinol stents Acta Radiol 2010 Sep 51 7 768 774 10.3109/02841851.2010.491093 20707660

[B16] Bajwa SJ Sehgal V Anesthesia and thyroid surgery: the never ending challenges Indian J Endocrinol Metab 2013 Mar 17 2 228 234 10.4103/2230-8210.109671 23776893 PMC3683195

[B17] Badr EM Osama YD The role of endoluminal self-expanding stents in the management of pediatric tracheal stenosis Int J Pediatr Otorhinolaryngol 2008 72 1371 1376 10.1016/j.ijporl.2008.05.015 18606458

[B18] Saad CP Murthy S Krizmanich G Mehta AC Self-expandable metallic airway stents and flexible bronchoscopy: long-term outcomes analysis Chest 2003 124 1993 1999 10.1378/chest.124.5.1993 14605078

[B19] Walser EM Stent placement for tracheobronchial disease Eur J Radiol 2005 55 321 330 10.1016/j.ejrad.2005.03.005 15913937

[B20] Chung F-T Chen H-C Chou C-L Chih-Teng Y An outcome analysis of self-expandable metallic stents in central airway obstruction: a cohort study J Cardiothorac Surg 2011 6 46 54 10.1186/1749-8090-6-46 21477303 PMC3090328

[B21] Tanigawa N Kariya S Komemushi A Nakatani M Metallic stent placement for malignant airway stenosis Minim Invasive Ther Allied Technol 2012 Mar 21 2 108 112 10.3109/13645706.2011.566272 21417832

[B22] Furukawa K Ishida J Yamaguchi G Usuda J The role of airway stent placement in the management of tracheobronchial stenosis caused by inoperable advanced lung cancer Surg Today 2010 Apr 40 4 315 320 10.1007/s00595-008-4058-2 20339985

[B23] Tanigawa N Sawada S Okuda Y Kobayashi M Symptomatic improvement in dyspnea following tracheobronchial metallic stenting for malignant airway obstruction Acta Radiol 2000 41 425 428 11016759 10.1080/028418500127345857

[B24] Chow DC Komaki R Libshitz HI Mountain CF Treatment of primary neoplasms of the trachea.The role of radiation therapy Cancer 1993 71 2946 2952 10.1002/1097-0142(19930515)71:10<2946::AID-CNCR2820711010>3.0.CO;2-E 8490822

[B25] Francisco VM Stephanie MA Ricardo LC Endoscopically placed expandable metal tracheal stents for the management of complicated tracheal stenosis Am J Otolaryngol 2003 24 34 40 10.1053/ajot.2003.6 12579481

